# MicroRNA-146a Decreases High Glucose/Thrombin-Induced Endothelial Inflammation by Inhibiting NAPDH Oxidase 4 Expression

**DOI:** 10.1155/2014/379537

**Published:** 2014-09-14

**Authors:** Huang-Joe Wang, Yuan-Li Huang, Ya-Yun Shih, Hsing-Yu Wu, Ching-Tien Peng, Wan-Yu Lo

**Affiliations:** ^1^School of Medicine, China Medical University, No. 91, Hsueh-Shih Road, Taichung 40402, Taiwan; ^2^Division of Cardiology, Department of Medicine, China Medical University Hospital, No. 2, Yuh-Der Road, Taichung 40447, Taiwan; ^3^Department of Biotechnology, College of Medical and Health Science, Asia University, No. 500, Lioufeng Road, Wufeng, Taichung 41354, Taiwan; ^4^Graduate Institute of Basic Medical Science, China Medical University, No. 91, Hsueh-Shih Road, Taichung 40402, Taiwan; ^5^Department of Life Science, National Chung Hsing University, No. 250, Kuo-Kuang Road, Taichung 40227, Taiwan; ^6^Graduate Integration of Chinese and Western Medicine, China Medical University, No. 91, Hsueh-Shih Road, Taichung 40402, Taiwan; ^7^Department of Pediatrics, Children's Hospital, China Medical University Hospital, No. 2, Yuh-Der Road, Taichung 40447, Taiwan; ^8^Department of Biotechnology, Asia University, No. 500, Lioufeng Road, Wufeng, Taichung 41354, Taiwan

## Abstract

Diabetes is associated with hyperglycemia and increased thrombin production. However, it is unknown whether a combination of high glucose and thrombin can modulate the expression of NAPDH oxidase (Nox) subtypes in human aortic endothelial cells (HAECs). Moreover, we investigated the role of a diabetes-associated microRNA (miR-146a) in a diabetic atherothrombosis model. We showed that high glucose (HG) exerted a synergistic effect with thrombin to induce a 10.69-fold increase in Nox4 mRNA level in HAECs. Increased Nox4 mRNA expression was associated with increased Nox4 protein expression and ROS production. Inflammatory cytokine kit identified that the treatment increased IL-8 and IL-6 levels. Moreover, HG/thrombin treatment caused an 11.43-fold increase of THP-1 adhesion to HAECs. *In silico* analysis identified the homology between miR-146a and the 3′-untranslated region of the Nox4 mRNA, and a luciferase reporter assay confirmed that the miR-146a mimic bound to this Nox4 regulatory region. Additionally, miR-146a expression was decreased to 58% of that in the control, indicating impaired feedback restraint of HG/thrombin-induced endothelial inflammation. In contrast, miR-146a mimic transfection attenuated HG/thrombin-induced upregulation of Nox4 expression, ROS generation, and inflammatory phenotypes. In conclusion, miR-146a is involved in the regulation of endothelial inflammation via modulation of Nox4 expression in a diabetic atherothrombosis model.

## 1. Introduction

Diabetes mellitus is an epidemic that affects all age groups worldwide. Both microvascular (nephropathy, neuropathy, and retinopathy) and macrovascular diseases (atherosclerosis of coronary, cerebral, and peripheral arteries) are prevalent in diabetic patients. Diabetes is associated with polyvascular, accelerated, and diffuse atherosclerotic diseases [1]. In advanced stages, the chances of atherosclerotic plaque rupture increase, which can cause atherothrombosis with clinical presentations of acute ischemic stroke, acute myocardial infarction, and peripheral arterial occlusive disease.

Endothelial dysfunction is an initiator of diabetic vascular diseases. Multiple diabetes-associated stresses, including hyperglycemia, impaired insulin signaling, advanced glycated end products, and dyslipidemia, lead to the inflammation of the endothelium, which can lead to thrombosis [2]. These metabolic abnormalities in diabetes increase the generation of reactive oxygen species (ROS), effectors that play a major role in producing a proinflammatory phenotype that culminates into accelerated atherosclerosis and atherothrombosis [3]. The most important ROS-generating systems in the vasculature include the mitochondrial electron transport chain, xanthine oxidase, and NADPH oxidases (Noxes) [2]. Noxes are transmembrane electron transporters that transport the cytoplasmic electron donor NADPH to oxygen in order to generate superoxide. Among the 7 known subtypes, Nox1, Nox2, Nox4, and Nox5 are expressed in endothelial cells and are the major sources of ROS in the cardiovascular systems [4].

MicroRNAs (miRs) are endogenous ~22-nucleotide RNAs that bind to the 3′-untranslated region (UTR) of a messenger RNA (mRNA) target. These miRs can regulate the oxidative and inflammatory state at the posttranscriptional level by causing degradation of the target mRNA or inhibiting translation [5]. Among the many miRs, miR-146a is identified as a negative regulator of NF-*κ*B, which targets IRAK1, TRAF6, and IRAK2 [6, 7]. Generally, an increased level of cellular miR-146a that exerts a negative feedback effect on NF-*κ*B signaling is observed during multiple stresses (e.g., TNF-*α*, LPS, interleukin- (IL-) 1*β*, PMA, and ox-LDL) [6, 8–11]. However, decreased cellular miR-146a levels have been reported in multiple diabetes-related studies, including in high glucose- (HG-) stimulated endothelial cells and diabetic rats [12, 13], in diabetic mouse wounds [14], in glycated albumin-stimulated endothelial cells [15], and in type 2 diabetes patients with subclinical inflammation and insulin resistance [16]. All of the above* in vitro* and* in vivo* evidences indicate that impaired miR-146a expression contributes to the proinflammatory state in diabetes.

The hallmark of diabetes is hyperglycemia. In addition, diabetes can cause a hypercoagulable state that is due to increased thrombin generation [17, 18]. Since HG and thrombin are known to increase ROS production [19, 20], the present study investigated whether a combination of HG and thrombin (HG/thrombin) influenced Nox subtype expression and endothelial inflammation. In addition, we tested whether miR-146a is involved in the regulation of Nox4 expression in this* in vitro* model of diabetic atherothrombosis.

## 2. Materials and Methods

### 2.1. Cell Culture

Human aortic endothelial cells (HAECs) were purchased from Cell Applications, Inc. (San Diego, CA) and cultured in endothelial cell growth medium (Cell Applications, Inc.) according to the manufacturer's recommendations. The cells were grown to near confluence in a 75-T flask before the experiment. All chemicals were obtained from Sigma-Aldrich (St. Louis, MO) unless otherwise specified. Thrombin at a dose of 2 units/mL and D-glucose (25 mmol/L) were added to the HAECs for the respective times. L-glucose (25 mmol/L) was used as an osmotic control. The human monocytic cell line THP-1 was obtained from the American Type Culture Collection (Rockville, MD) and maintained in RPMI-1640 culture medium (Thermo Scientific HyClone, Logan, UT) supplemented with 10% FBS and 1% penicillin-streptomycin (Invitrogen, Carlsbald, CA).

### 2.2. Real-Time Polymerase Chain Reaction (PCR)

The mRNA expression level in the HAECs was analyzed by real-time PCR, as previously described [21]. The glyceraldehyde-3-phosphate dehydrogenase (GAPDH) gene was selected for normalization of data. These real-time PCR data were calculated by the 2^−ΔΔCt^ method for RNA quantification. The primer sequences used for the amplification of genes encoding* GAPDH*,* Nox1*,* Nox2*,* Nox4*,* Nox5*, interleukin-6 (*IL-6*), interleukin-8 (*IL-8*), vascular cell adhesion protein 1 (*VCAM-1*), intercellular adhesion molecule 1 (*ICAM-1*), and E-selectin (*SELE*) were as follows:

 
*GAPDH*:
 Forward primer: 5′-CTCTGCTCCTCCTGTTCGAC-3′
 Reverse primer: 5′-ACGACCAAATCCGTTGACTC-3′
 
*Nox1*:
 Forward primer: 5′-TCACCCCCTTTGCTTCTATCT-3′
 Reverse primer: 5′-AATGCTGCATGACCAACCTT-3′
 
*Nox2*:
 Forward primer: 5′-CATTCAACCTCTGCCACCAT-3′
 Reverse primer: 5′-CCCCAGCCAAACCAGAAT-3′
 
*Nox4*:
 Forward primer: 5′-GCTGACGTTGCATGTTTCAG-3′
 Reverse primer: 5′-CGGGAGGGTGGGTATCTAA-3′
 
*Nox5*:
 Forward primer: 5′-GGAGAAGATGAACACATCTGGAG-3′
 Reverse primer: 5′-CATCCTCCTCGGCACTCA-3′
 
*IL-6*:
 Forward primer: 5′-GATGAGTACAAAAGTCCTGATCCA-3′
 Reverse primer: 5′-CTGCAGCCACTGGTTCTGT-3′
 
*IL-8*:
 Forward primer: 5′-AGACAGCAGAGCACACAAGC-3′
 Reverse primer: 5′-ATGGTTCCTTCCGGTGTT-3′
 
*VCAM-1*:
 Forward primer: 5′-TGCACAGTGACTTGTGGACAT-3′
 Reverse primer: 5′-CCACTCATCTCGATTTCTGGA-3′
 
*ICAM-1*:
 Forward primer: 5′-CCTTCCTCACCGTGTACTGG-3′
 Reverse primer: 5′-AGCGTAGGGTAAGGTTCTTGC-3′
 
*SELE*:
 Forward primer: 5′-ACCAGCCCAGGTTGAATG-3′
 Reverse primer: 5′-GGTTGGACAAGGCTGTGC-3′

### 2.3. Western Blot Analysis

The protein expression level in the HAECs was analyzed by western blot analysis, as previously described [21]. Antibodies against Nox4 (GeneTex, Irvine, CA) and GAPDH (Santa Cruz Biotechnology, Santa Cruz, CA) were used at concentrations of 1 : 1000 and 1 : 2000, respectively. Whole cell lysates were used in all blots, which were normalized to GAPDH.

### 2.4. Intracellular ROS Determination

Intracellular ROS expression level was analyzed by an Oxiselect Intracellular ROS Assay Kit (Cell Biolabs, San Diego, CA), which measures ROS by employing the cell-permeable fluorogenic probe 2′,7′-dichlorodihydrofluorescein diacetate (DCFH-DA). Briefly, HAECs (1 × 10^4^) were stimulated with different stressors for 5 h before determining the level of ROS. The level of ROS was determined according to the manufacturer's protocol. In separate experiments, the HAECs were transfected with a miR-146a mimic before determining ROS production.

### 2.5. Inflammatory Cytokine Expression

HAECs were plated onto coverslips in a 24-well plate at a density of 5 × 10^5^ cells/well and stimulated with thrombin and/or HG. After treatment, the supernatants were collected and separated by centrifugation at 500 ×g for 5 min at 4°C and stored at −80°C until used for analysis. A panel of cytokines including IL-8, IL-1*β*, IL-6, IL-10, TNF-*α*, and IL-12p70 was simultaneously examined using the Human Inflammatory Cytokines Kit (BD Biosciences Pharmingen, San Diego, CA) through flow cytometry (BD FACSCanto system; Becton Dickinson Corp., San Jose, CA), as previously described [15].

### 2.6. Monocyte Adhesion Assay

In adhesion experiments, THP-1 cells were labeled with calcein acetoxymethyl ester (Calcein-AM; Molecular Probes, OR). The THP-1 cells were stained with the dye at a concentration of 7.5 *μ*M for 30 min immediately preceding the adhesion assay. HAECs were maintained in 12 wells until 90% confluence. The HAECs (10^5^ cells/well) were then treated with thrombin and/or HG for 5 h and incubated with culture medium containing the labeled THP-1 cells (THP-1/HAECs = 7) for 10 min. After incubation, nonadherent THP-1 cells were removed by gentle washing with PBS for 20 s. The adherent THP-1 cells on different thrombin and/or HG-activated ECs were identified and quantified in 10 randomly selected view fields. In separate experiments, the THP-1 adhesion assay was performed after miR-146a mimic transfection.

### 2.7. miR Extraction and Analysis

The protocols for miR extraction and determination of miR-146a expression level were previously described [15]. RNU6B expression level served as a loading control.

### 2.8. Luciferase Reporter Assay

The partial Nox-4 mRNA 3′-UTR containing the miR-146a target site was constructed into a pGL-2-promoter vector (Promega, Madison, WI). HAECs were cotransfected with 1 *μ*g of constructed plasmids and 100 nM of miR-146a mimic or miR-control sequences using Lipofectamine 2000 (Invitrogen, Carlsbad, CA). After 24 h of transfection, cells were harvested to measure luciferase activity according to the manufacturer's procedures (Promega).

### 2.9. miR-146a Mimic Transfection

The miR-146a mimic (mirVana miRNA mimic) and miR-control were obtained from Ambion (Austin, TX). HAECs were transfected with hsa-miR-146a mimic (30 nM) or miR-control (30 nM) using the Lipofectamine 2000 transfection reagent (Invitrogen) in serum-free M-199 medium for 2 h. After transfection, the M-199 medium was changed to the endothelial cell growth medium. HAECs were then treated with thrombin and HG for different times in the respective experiments. After treatment, the supernatants were collected and preserved for studies of inflammatory cytokine expression. The HAECs were collected, and the expression levels of* Nox4*,* VCAM-1*,* ICAM-1*,* SELE*,* IL6*,* IL8* mRNA, Nox4 protein, and ROS activity were determined. In addition, THP-1 adhesion experiments were performed after miR-146a mimic transfection.

### 2.10. Statistical Analysis

Statistical analyses were performed using SPSS 12.0 statistical software for Windows (SPSS Inc., Chicago, IL). All data are presented as the mean ± SEM. The significance between 2 groups was determined using unpaired *t*-tests. *P* values less than 0.05 were considered statistically significant.

## 3. Results

### 3.1. Endothelial* Nox4* and Intracellular ROS Were Significantly Induced by HG/Thrombin

**Figure 1:**
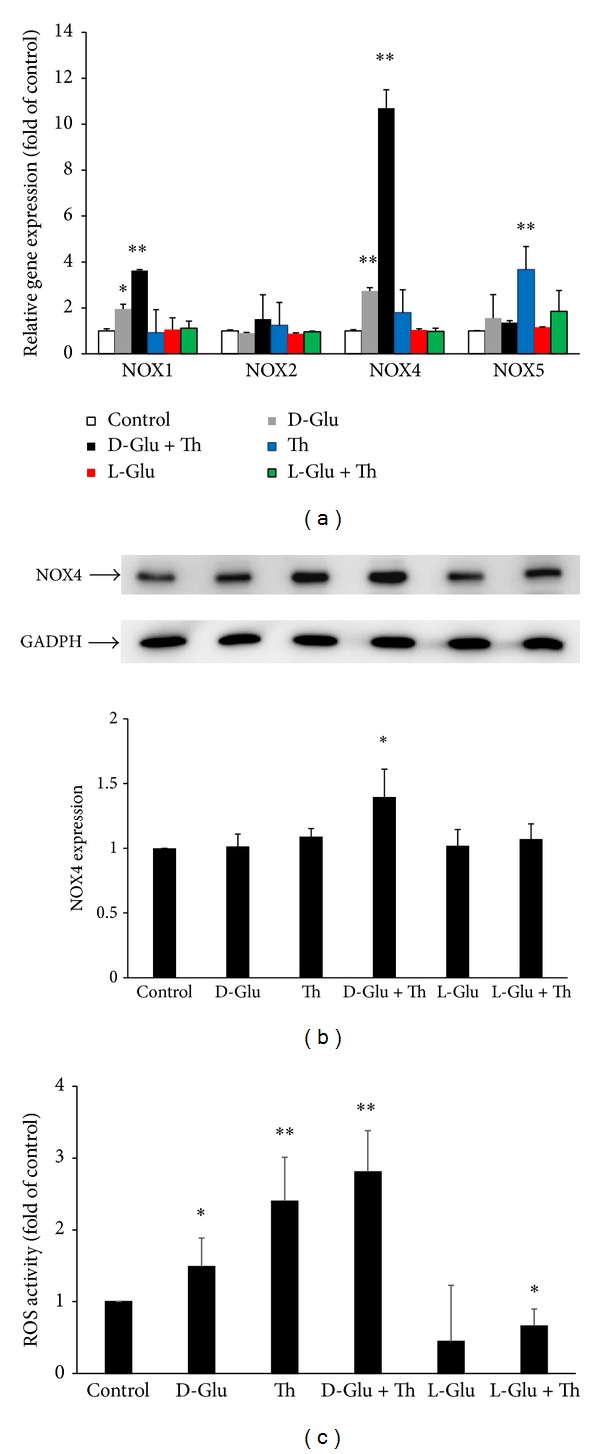
Endothelial Nox4 expression and intracellular ROS production were significantly induced by HG/thrombin. (a) HAECs were stimulated with HG (25 mmol/L), L-glucose (25 mmol/L), and thrombin (2 U/mL) for 5 h. Real-time PCR showed that stimulation of HAECs with D-glucose/thrombin induced a 10.69-fold synergistic increase of* Nox4* mRNA levels compared with the control, while L-glucose/thrombin had no such effect. The bars represent the means ± SEM from 3 experiments. *P < 0.05, as compared with the control. **P < 0.01, as compared with the control. (b) Stimulation of HAECs with HG/thrombin for 12 h increased Nox4 protein expression 1.35-fold compared with the control; alternatively, L-glucose/thrombin had no discernible effect on Nox4 expression. The bars represent the means ± SEM from 4 experiments. *P < 0.05, as compared with the control. (c) Stimulation of HAECs with HG/thrombin for 5 h significantly increased ROS production 2.81-fold compared with the control. The bars represent the means ± SEM from 3 experiments. *P < 0.05, as compared with the control. **P < 0.01, as compared with the control.

We first examined the effects of HG and thrombin on the expression of endothelial* Nox1*,* Nox2*,* Nox4*, and* Nox5* mRNA expression (Figure 1(a)). After 5 h of stimulation, high D-glucose (HG, 25 mmol/L) alone caused significant 1.95- and 2.74-fold increases in* Nox1* and* Nox4 *mRNA expression, respectively, while the* Nox2* and* Nox5* mRNA expression did not change. Thrombin (2 U/mL) alone caused a significant 3.67-fold increase in* Nox5* mRNA expression, while* Nox1*,* Nox2*, and* Nox4* mRNA expression did not change. HG/thrombin cotreatment caused significant 3.62- and 10.69-fold increases in* Nox1* and* Nox4 *mRNA, respectively, while* Nox2* and* Nox5* expression did not change. Both L-glucose treatment and L-glucose/thrombin cotreatment had no discernible effects on the mRNA expression of* Nox1*,* Nox2*,* Nox4*, and* Nox5*.

In the western blot analysis, HG/thrombin cotreatment for 12 h caused a significant 1.33-fold increase in Nox4 expression compared with the control, while the L-glucose treatment and L-glucose/thrombin cotreatment had no discernible effects on the protein expression of Nox4 (Figure 1(b)). To determine if HG/thrombin-induced Nox4 expression was associated with enhanced intracellular ROS production, DCFH-DA assay was employed. As shown in Figure 1(c), HG and thrombin treatment alone for 5 h caused significant 1.48- and 2.40-fold increases of ROS production in HAECs, respectively. HG/thrombin cotreatment further increased the ROS production up to 2.81-fold.

### 3.2. Endothelial Inflammation Was Significantly Induced by HG/Thrombin

**Figure 2:**
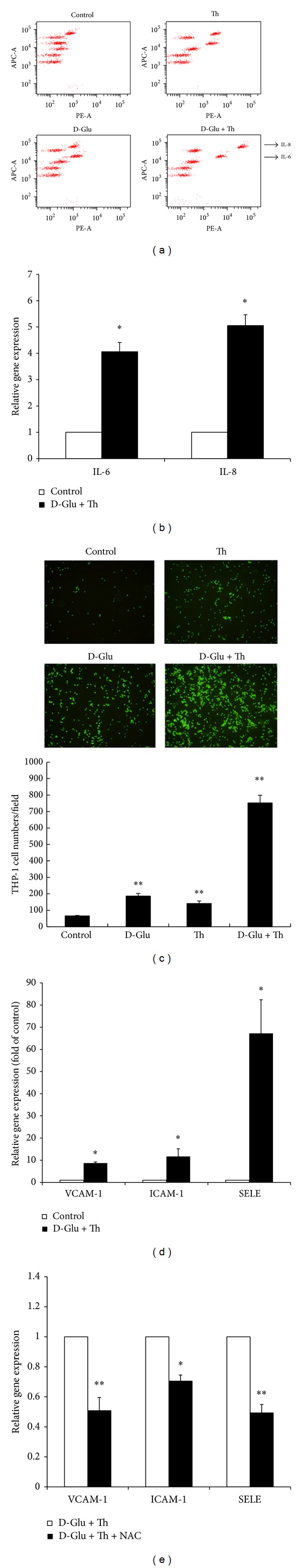
Endothelial inflammation was induced by HG/thrombin through an ROS-dependent pathway. (a) From top to bottom, multiple small dots accumulated into 6 horizontal lines, which represented the expression levels of IL-8, IL-1*β*, IL-6, IL-10, TNF-*α*, and IL-12p70 proteins in conditioned medium. The rightward shift of the dotted lines represents the relative protein expression levels. Analysis using an inflammatory cytokine kit showed that HG/thrombin stimulation increased IL-8 and IL-6 expression in HAECs, while L-1*β*, IL-10, TNF-*α*, and IL-12p70 had no discernible effect on its protein expression levels. The images shown represent the results from 3 independent experiments. (b) Stimulation of HAECs with HG/thrombin for 5 h increased caused 4.06-fold and 5.05-fold increases of* IL-6* and* IL-8* mRNA gene expression levels, respectively. The bars represent the means ± SEM from 3 experiments. *P < 0.05, as compared with the control. (c) Stimulation of HAECs with HG/thrombin for 5 h induced an 11.43-fold synergistic increase of THP-1 adhesiveness to HAECs compared with the control. The bars represent the means ± SEM from 4 experiments. **P < 0.01, as compared with the control. (d) Stimulation of HAECs with HG/thrombin for 5 h induced significant 8.72-, 11.67-, and 67.21-fold increases of* VCAM-1*,* ICAM-1*, and* SELE* gene expression, respectively. The bars represent the means ± SEM from 3 experiments. *P < 0.05, as compared with the control. (e) HAECs were pretreated with 5 mM N-acetylcysteine (NAC) for 1 h and then stimulated with HG/thrombin for 5 h. Real-time PCR showed that NAC pretreatment caused significant decreases in the gene expression of* VCAM-1*,* ICAM-1*, and* SELE *to 51%, 70%, and 49% of that in the HG/thrombin-stimulated HAECs. The bars represent the means ± SEM from 4 experiments. *P < 0.05 and **P < 0.01, as compared with the HG/thrombin.

HG/thrombin-induced inflammatory cytokine changes were first examined using the Human Inflammatory Cytokines Kit. The HG/thrombin cotreatment for 12 h caused an increase in the IL-8 and IL-6 levels in the conditioned medium, as shown by the rightward shift of the dotted lines; however, IL-1*β*, IL-10, TNF-*α*, and IL-12p70 expression levels were not affected (Figure 2(a)). In addition, HG/thrombin cotreatment caused 4.06-fold and 5.05-fold increases of* IL-6* and* IL-8 *mRNA gene expression levels, respectively (Figure 2(b)).

The adhesion of circulating monocytes to endothelial cells is an important event in causing vascular inflammation and atherosclerosis development [22]. As shown in Figure 2(b), HG/thrombin cotreatment significantly increased monocytic THP-1 cell adhesion to HAECs. While HG and thrombin treatment alone for 5 h caused 2.84- and 2.15-fold increases in the THP-1 adhesion to HAECs, respectively, HG/thrombin cotreatment caused an 11.43-fold synergistic increase of THP-1 adhesion (Figure 2(c)). The increased THP-1 adhesiveness was associated with increased gene expression of cellular adhesion molecules (CAMs). HG/thrombin cotreatment caused 8.72-fold, 11.67-fold, and 67.21-fold increases of* VCAM-1*,* ICAM-1*, and* SELE* mRNA gene expression levels, respectively (Figure 2(d)). These data demonstrate that increased expression of these CAMs was at least partially responsible for increased THP-1 adhesiveness in HG/thrombin-activated HAECs.

Oxidative stress is known to impair endothelial function. Therefore, we tested whether ROS are involved in the HG/thrombin-induced endothelial inflammation. As shown in Figure 2(e), an ROS scavenger N-acetylcysteine (NAC, 5 mM) pretreatment caused a significant decrease in the gene expression of* VCAM-1*,* ICAM-1*, and* SELE*, suggesting that ROS are involved in the HG/thrombin-induced endothelial inflammation.

### 3.3. *Nox4* Is a Direct Target of miR-146a

**Figure 3:**
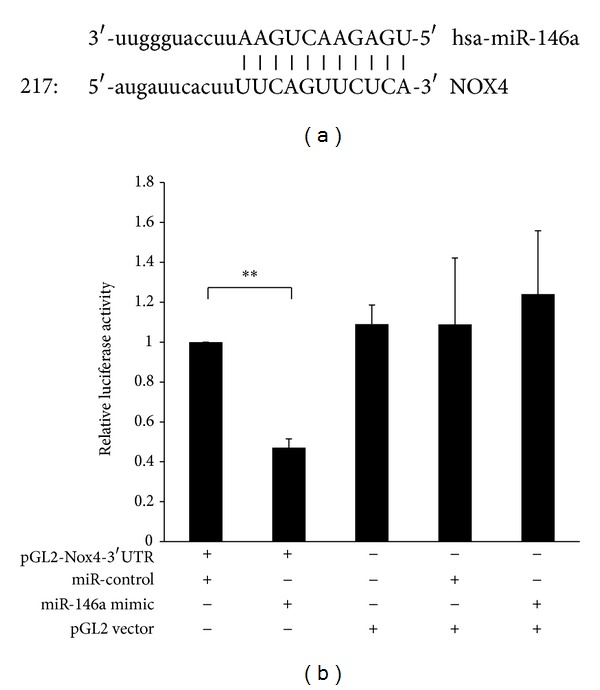
Nox4 was identified as a target of miR-146a. (a) Bioinformatic miR target analysis identified homology between miR-146a and the 3′-UTR of the human Nox4 mRNA, indicating potential regulation of Nox4 by miR-146a. (b) A luciferase reporter assay showed that the miR-146a mimic could downregulate relative luciferase activity of pGL2-Nox4-3′-UTR compared with the miR-control. The bars represent the means ± SEM from 6 experiments. **P < 0.01, as compared with the miR-control.

Bioinformatic miR target analysis using miR databases (http://www.microrna.org/microrna/getGeneForm.do) identified homology between miR-146a and the 3′-UTR of the human Nox4 mRNA, indicating that* Nox4* is a potential miR-146a target (Figure 3(a)). To investigate whether miR-146a can interact with Nox4 mRNA 3′-UTR, a luciferase reporter assay was performed. As shown in Figure 3(b), cotransfection of pGL2-Nox4-3′-UTR and the miR-146a mimic resulted in a decrease in luciferase signal to 47% of that in the miR-control, which confirmed direct binding of miR-146a to the Nox4 3′-UTR.

### 3.4. HG/Thrombin Downregulated miR-146a Expression

**Figure 4:**
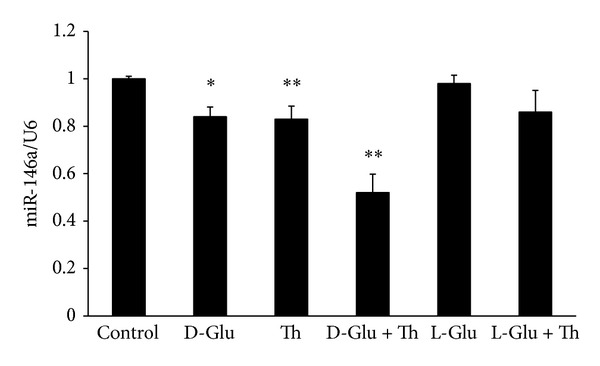
HG/thrombin downregulated miR-146a expression in HAECs. miR-146a expression was measured by real-time PCR. HG/thrombin stimulation for 5 h decreased miR-146a expression to 58% of that in the control. The bars represent the means ± SEM from 5 experiments. *P < 0.05 and **P < 0.01, as compared with the control. miR levels are expressed as the ratio of RNU6B levels.

Since miR-146a is an important diabetes-associated posttranscriptional regulator, we determined whether miR-146a levels changed in HG/thrombin-stimulated HAECs. As shown in Figure 4, treatment with either HG or thrombin separately for 5 h significantly decreased miR-146a expression to 83% and 76% of that in the control, respectively. HG/thrombin cotreatment further downregulated the miR-146a expression to 58% of that in the control. In contrast, both L-glucose treatment and L-glucose/thrombin cotreatment had no discernible effects on the expression of miR-146a. These findings indicate that the anti-inflammatory feedback circuit mediated by cellular miR-146a levels was impaired in the HG/thrombin-activated HAECs.

### 3.5. *Nox4* Expression and Intracellular ROS Production Were Attenuated in miR-146 Mimic-Transfected, HG/Thrombin-Treated HAECs

**Figure 5:**
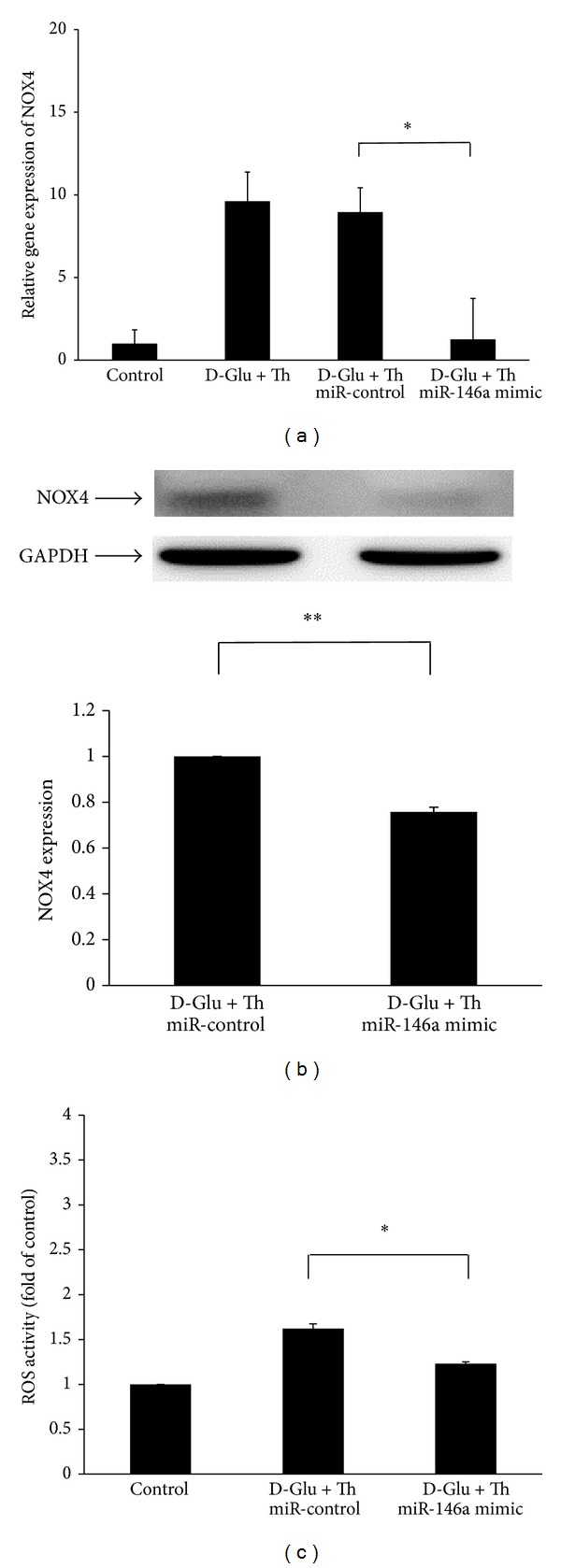
The stimulatory effects of HG/thrombin on Nox4 expression and ROS production were attenuated in miR-146a mimic-transfected HAECs. (a) The stimulatory effect of HG/thrombin on* Nox4* mRNA expression was preserved in miR-control-transfected HAECs, with an increase of 8.95-fold* Nox4* mRNA levels compared with the control. In contrast, HG/thrombin did not increase* Nox4* mRNA expression in miR-146a mimic-transfected HAECs. The bars represent the means ± SEM from 3 experiments. *P < 0.05, as compared with the miR-control. (b) The stimulatory effect of HG/thrombin on Nox4 protein expression was significantly attenuated in miR-146a mimic-transfected HAECs. The bars represent the means ± SEM from 4 experiments. **P < 0.01, as compared with the miR-control. (c) The stimulatory effect of HG/thrombin on intracellular ROS production was significantly attenuated in miR-146a mimic-transfected, HG/thrombin-stimulated HAECs. The bars represent the means ± SEM from 3 experiments. *P < 0.05, as compared with the miR-control.

To determine whether miR-146a can inhibit Nox4 expression in the HG/thrombin-stimulated HAECs, we transfected HAECs with a miR-146a mimic. Regarding* Nox4* mRNA expression, the stimulatory effect of HG/thrombin was preserved in the miR-control-transfected, HG/thrombin-stimulated HAECs. In contrast, the miR-146a mimic abolished the stimulatory effect of* Nox4* mRNA expression in HG/thrombin-stimulated HAECs (Figure 5(a)). Regarding Nox4 protein expression, the stimulatory effect of HG/thrombin on Nox4 protein expression was also attenuated in miR-146a mimic-transfected HAECs (Figure 5(b)). This inhibitory effect of the miR-146 mimic on the Nox4 expression concurrently occurred with decreased ROS production in the HG/thrombin-stimulated HAECs (Figure 5(c)).

### 3.6. Endothelial Inflammation Was Attenuated by the miR-146 Mimic

**Figure 6:**
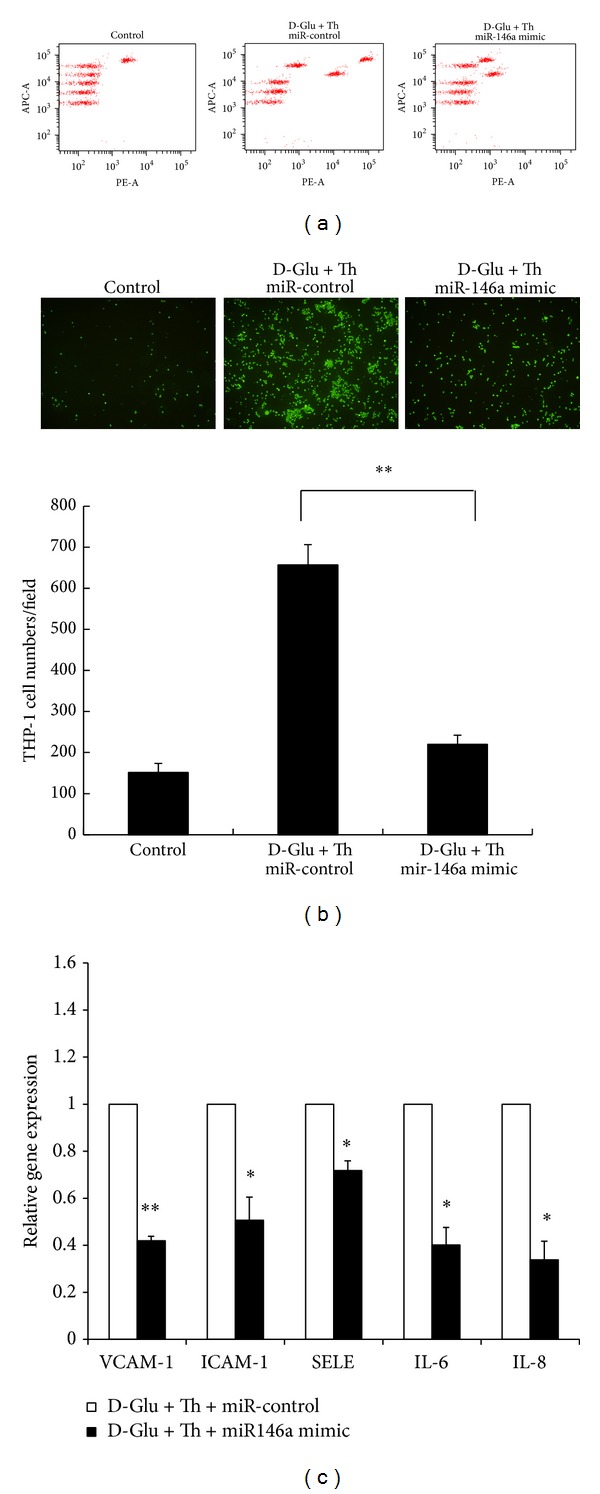
The proinflammatory phenotypes of HG/thrombin-stimulated HAECs were attenuated by a miR-146a mimic. (a) Analysis using an inflammatory cytokine kit showed that the stimulatory effect of HG/thrombin on IL-8 and IL-6 protein expression in conditioned mediums was preserved in miR-control-transfected HAECs, while the stimulatory effect was attenuated in miR-146a mimic-transfected, HG/thrombin-stimulated HAECs. The images shown represent the results from 3 independent experiments. (b) The stimulatory effect of HG/thrombin on THP-1 adhesion to HAECs was preserved in miR-control-transfected HAECs, while the THP-1 adhesiveness was attenuated in miR-146a mimic-transfected, HG/thrombin-stimulated HAECs to 34% of that in the miR-control-transfected HACEs. The bars represent the means ± SEM from 4 experiments. **P < 0.01, as compared with the miR-control. (c) The stimulatory effect of HG/thrombin on the gene expression of* VCAM-1*,* ICAM-1*,* SELE*,* IL-6*, and* IL-8* significantly decreased in miR-146a mimic-transfected, HG/thrombin-stimulated HAECs to 42%, 51%, 71%, 40%, and 34% of that in miR-control-transfected, HG/thrombin-stimulated HAECs. The bars represent the means ± SEM from 3 experiments. *P < 0.05 and **P < 0.01, as compared with the miR-control.

To further determine whether the reduced Nox4 expression and ROS production were associated with reduced endothelial inflammation in the miR-146 mimic-transfected, HG/thrombin-stimulated HAECs, we measured inflammatory cytokine expression, THP-1 adhesion, and inflammatory gene expression. As anticipated, both IL-8 and IL-6 protein expression in the conditioned medium were attenuated by miR-146a mimic transfection (Figure 6(a)). Furthermore, the adhesion of THP-1 cells (Figure 6(b)) and gene expression of* VACM-1*,* ICAM-1*,* SELE*,* IL-6*, and* IL-8* (Figure 6(c)) were also significantly reduced in the miR-146a transfected, HG/thrombin-stimulated HAECs.

**Figure 7:**
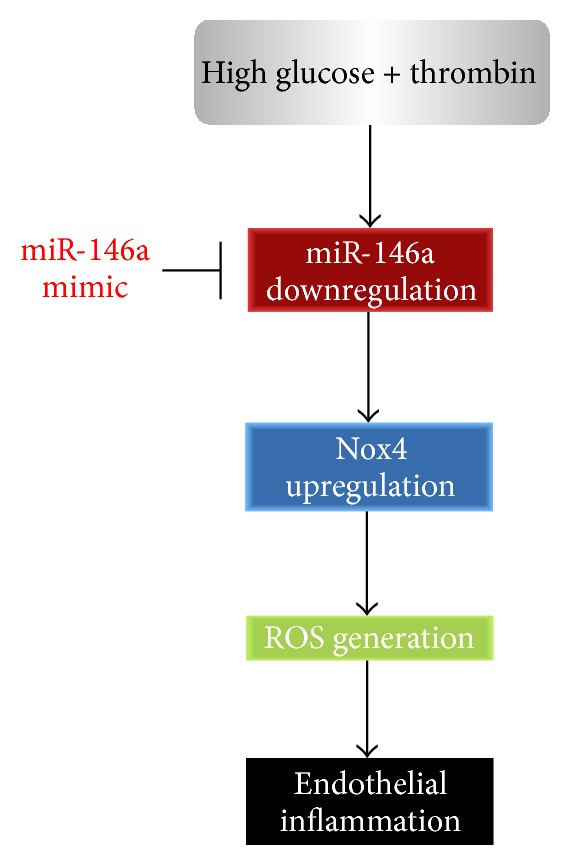
The proposed role of miR-146a in regulating HG/thrombin-induced endothelial inflammation.

The proposed role of miR-146a in regulating HG/thrombin-induced endothelial inflammation is shown in Figure 7.

## 4. Discussion

This study demonstrated that HG/thrombin exerts a synergistic effect to upregulate* Nox4* mRNA expression, Nox4 protein expression and to increase ROS generation in HAECs. Such diabetes-associated stimuli also downregulate endothelial miR-146a cellular level and enhance endothelial inflammatory phenotypes. The luciferase reporter assay and transfection experiments confirm the role of miR-146a in the posttranscriptional regulation of Nox4 expression. Furthermore, overexpression of the miR-146a mimic can result in a vascular protective effect by its anti-inflammatory effect in HG/thrombin-stimulated HAECs. Such properties of miR-146a may be useful for treating accelerated atherosclerosis and atherothrombosis in diabetes.

Blood vessels expressed a high level of Nox4 [4]. Additionally, gene expression studies reported that* Nox4* was the dominant isoform expressed in endothelial cells [23, 24]. In endothelial cells, multiple cellular stresses, including oscillatory shear stress [25], TNF-*α* [26], lipopolysaccharide [27], oxidized-phospholipids [28], and glycated albumin [29], were reported to upregulate endothelial Nox4 expression. The upregulated Nox4, in turn, mediated multiple detrimental effects on endothelial cells, including senescence [30], apoptosis [31], and proinflammatory response [27]. Elevated Nox4 expression was previously reported in many cardiovascular diseases including atherosclerosis, pulmonary artery hypertension, stroke, and heart failure [32]. In addition, Nox4 plays a critical role in mediating diabetic nephropathy [33] and diabetic retinopathy [34]. Our data provide evidence that Nox4 expression, ROS production, and endothelial inflammation were strongly upregulated in the HG/thrombin-stimulated HAECs, further supporting Nox4-mediated vascular complications in diabetes. Interestingly, although the L-glucose alone did not change endothelial* Nox4* expression, it did cause reduction of thrombin-induced* Nox5* mRNA expression and ROS production. Valeri et al. reported that both fructose and D-tagatose (L-epimer of D-fructose) had cytoprotective effects in prooxidant (i.e., cocaine or nitrofurantoin) induced rat hepatocyte cell injury; these effects were exerted by suppressing the iron-catalyzed formation of ROS [35]. Moreover, Paterna et al. also showed that D-tagatose prevented oxidative cell injury induced by nitrofurantoin or toxic iron overload with ferric nitrilotriacetate in mouse hepatocytes [36]. Their findings revealed that other monosaccharides might have a role in attenuating oxidative cell injury, although additional studies are needed to address the role of L-glucose in alleviating ROS production.

Nox4 activity is constitutive for its ROS-generating ability [37]. The close correlation between ROS generation and* Nox4* mRNA level indicates that* Nox4 *is an inducible isoform [38]. As shown in our data, the amount of ROS production and the degree of* Nox4* mRNA upregulation indicate that increased ROS production in HG/thrombin-stimulated HAECs was at least partially dependent on* Nox4 *upregulation. ROS are important signaling molecules that participate in endothelial inflammation [39]. Previous studies reported that Nox4 played an important role in mediating IL-8 expression [27] and THP-1 adhesion [40, 41]. Consistent with these findings, we observed attenuated ROS production and endothelial inflammation after downregulation of Nox4 expression with a miR-146a mimic, indicating that Nox4-inducing ROS were partially responsible for the proinflammatory response in HG/thrombin-stimulated HAECs.

Nox4 is mostly regulated by transcription via several transcription factors, including E2F, NF*κ*B, STAT1/3, HIF-1*α*, Nrf2, and Oct-1 [42]. The posttranscriptional regulation of Nox4, however, is less well understood. Interestingly, 2 separate groups reported that Nox4 was regulated at the posttranscriptional level by miR-25. In the first study, Fu et al. reported that miR-25 was significantly reduced in HG-treated rat mesangial cells and the kidneys of streptozotocin-induced diabetic rats. These researchers demonstrated that miR-25 was a direct target of Nox4 3′-UTR and concluded that miR-25 may contribute to the regulation of Nox4 expression in diabetic nephropathy [43]. In the second group, Varga et al. demonstrated that miR-25 was downregulated in the heart of cholesterol-enriched diet-induced hypercholesterolemia rats. These researchers concluded that downregulated miR-25 contributes to myocardial oxidative/nitrative stress through increased Nox4 expression [44]. Similar to the regulation patterns of miR-25, we also noted that HG/thrombin downregulated miR-146a expression and upregulated Nox4 expression. A luciferase reporter assay confirmed that* Nox4 *3′-UTR is a direct target of miR-146a. In addition, transfection experiments further support that the stimulatory effect of HG/thrombin on Nox4 expression was at least partially mediated by a posttranscriptional regulatory mechanism via cellular miR-146a levels. All of our findings also indicate a role of impaired miR-146a expression in diabetes.

Previous studies have shown that miR-146a negatively modulated IL-6 and IL-8 in human fibroblasts [45] and human trabecular meshwork cells [46]. Our data also showed that the miR-146 mimic downregulated IL-6 and IL-8 expression in HG/thrombin-stimulated HAECs. Increased IL-6 expression and signaling events are known to contribute to both atherosclerotic plaque growth and plaque destabilization [47]. In diabetes, IL-6 was reported as an independent predictor of microvascular complications and cardiovascular disease [48]. On the other hand, IL-8 signaling was important for the angiogenic response and survival of endothelial cells [49]. Angiogenesis in atherosclerotic plaques is important for plaque development and plaque vulnerability [50]. Increased IL-8 levels are also reported to predict cardiovascular events in patients with stable coronary artery disease [51]. Our data demonstrate that both IL-6/IL-8 expression and THP-1 adhesion were strongly upregulated by HG/thrombin, indicating that diabetic plaque rupture with thrombin generation in a hyperglycemic medium can exert a vicious autoamplifying cycle by further accelerating vascular inflammation and atherosclerosis development.

CAMs (VCAM-1, ICAM-1, and SELE) are important for macrophage recruitment, endothelial inflammation, and subsequent atherosclerotic plaque development [22]. By activating the HAECs with HG/thrombin, we observed increased expression of these CAMs, while the miR-146a mimic attenuated expression of these CAMs and associated THP-1 adhesiveness. Interestingly, Wu et al. reported that aerobic exercise and statins could increase miR-146a levels in ApoE-null mice, thereby attenuating vascular inflammation through reducing TLR4 and TRAF6 signaling [52]. Our previous work also found that angiotensin-(1–7) can decrease endothelial IL-6 expression in glycated albumin-stimulated HAECs through its ability to enhance endogenous miR-146a levels [15]. All of these findings supported the beneficial effects of modulating miR-146a in atherosclerosis and diabetes-associated vascular injury.

## 5. Conclusions

HG/thrombin causes impairment of miR-146a expression with an increase of Nox4 expression, ROS production, and inflammatory phenotypes in HAECs. In contrast, the miR-146a mimic exerts an endothelial protective effect by attenuating Nox4 expression, ROS production, and endothelial inflammation. These findings indicate therapeutic potential of modulating miR-146a in alleviating diabetic vascular complications.
